# Neuronal adaptation, novelty detection and regularity encoding in audition

**DOI:** 10.3389/fnsys.2014.00111

**Published:** 2014-06-24

**Authors:** Manuel S. Malmierca, Maria V. Sanchez-Vives, Carles Escera, Alexandra Bendixen

**Affiliations:** ^1^Auditory Neurophysiology Unit, Laboratory for the Neurobiology of Hearing, Institute of Neuroscience of Castilla y León, University of SalamancaSalamanca, Spain; ^2^Department of Cell Biology and Pathology, Faculty of Medicine, University of SalamancaSalamanca, Spain; ^3^Institució Catalana de Recerca i Estudis Avançats (ICREA)Barcelona, Spain; ^4^Institut de Investigacions Biomèdiques August Pi i Sunyer (IDIBAPS)Barcelona, Spain; ^5^Institute for Brain, Cognition and Behavior (IR3C), University of BarcelonaBarcelona, Spain; ^6^Cognitive Neuroscience Research Group, Department of Psychiatry and Clinical Psychobiology, University of BarcelonaBarcelona, Spain; ^7^Auditory Psychophysiology Lab, Department of Psychology, Cluster of Excellence “Hearing4all”, European Medical School, Carl von Ossietzky University of OldenburgOldenburg, Germany

**Keywords:** auditory, potassium channels, regularity, deviance detection, sensory adaptation, stimulus-specific adaptation (SSA), mismatch negativity (MMN), middle latency response (MLR)

## Abstract

The ability to detect unexpected stimuli in the acoustic environment and determine their behavioral relevance to plan an appropriate reaction is critical for survival. This perspective article brings together several viewpoints and discusses current advances in understanding the mechanisms the auditory system implements to extract relevant information from incoming inputs and to identify unexpected events. This extraordinary sensitivity relies on the capacity to codify acoustic regularities, and is based on encoding properties that are present as early as the auditory midbrain. We review state-of-the-art studies on the processing of stimulus changes using non-invasive methods to record the summed electrical potentials in humans, and those that examine single-neuron responses in animal models. Human data will be based on mismatch negativity (MMN) and enhanced middle latency responses (MLR). Animal data will be based on the activity of single neurons at the cortical and subcortical levels, relating selective responses to novel stimuli to the MMN and to stimulus-specific neural adaptation (SSA). Theoretical models of the neural mechanisms that could create SSA and novelty responses will also be discussed.

## Introduction

Traditionally, studies on the auditory nervous system have relied on the analysis of neuronal responses to simple stimuli. While this approach has been useful to understand the basic mechanisms that operate in the auditory system, recent studies are using more ecologically valid stimuli to explore the interplay of the different levels of the auditory hierarchy, from the brainstem to the cortex, in a realistic environment. To navigate in an ever-changing real-life scene, the brain is continuously using the sensory past to form expectations about the future.

Most of us have experienced an unbearably loud clock or a busy street that—mysteriously—after a while we stop noticing. Adaptation is a common feature in sensory processing, and the auditory system is not an exception. There are different theories that are not mutually exclusive, about why sensory adaptation is so pervasive. It decreases the amount of information flowing in the system when the stimulus does not vary or is repeated, it increases the sensitivity to detect stimulus changes and abrupt onsets (Ulanovsky et al., 2003; Puccini et al., 2006) and/or it provides a gain control mechanism in the encoding system (Rabinowitz et al., 2011). All these strategies facilitate the detection of novel stimuli, making adaptation and novelty detection closely intertwined.

In order for the nervous system to determine whether a sound is “novel”—in its current context–, there must be some ongoing storage of information about which sounds have already occurred and how they are related to each other. Novelty or unexpectedness can arise from rareness or from abrupt change (as in the above examples), but can also take more complex forms: In some cases (such as when a sound source is moving away from the listener), stimulus change is expected while stimulus repetition is unexpected. The auditory system easily forms expectations at different levels of complexity (Näätänen et al., 2001, 2010); hence the related phenomena are regarded as signs of regularity encoding rather than mere adaptation to an unchanging environment (Winkler, 2007). In this perspective article, we will discuss the neuronal basis and functional implications of this amazing feat of the auditory system.

## Stimulus-specific adaptation appears subcortically

Human electroencephalographic (EEG) studies of responses to sensory stimuli have shown that the waveform elicited by a “novel” (low-probability) stimulus differs from that elicited by a predictable (high-probability) stimulus. Indeed, the detection of “novel” (unexpected) sounds has been associated to a particular brain response derived from the human EEG, the mismatch negativity (MMN; Näätänen et al., 1978; for recent review, see Näätänen et al., 2007). The MMN is an auditory event-related brain potential (ERP) component thought to index the mechanisms underlying auditory regularity encoding. MMN can be obtained using an “oddball paradigm”, in which a high-probability (“standard”) sound is occasionally replaced by a low-probability (“deviant”) sound. Differences in sound probability are created by setting up a sequential regularity; that is, a constraint in the allowed transitions between successive stimuli in the sequence. In the simplest case, this regularity is based on stimulus/feature repetition (cf. Grill-Spector et al., 2006, for a comprehensive account in the visual domain); more complex cases include feature alternation, gradual progression, or feature conjunctions (cf. Näätänen et al., 2001, 2010; Winkler, 2007). MMN is measured as the difference between the ERP elicited by the deviant sound and that elicited by the standard sound and peaks at 150–200 ms from deviation onset (Note that if the standard is defined by repetition, a compensation for the larger afferent responses of the infrequent deviant sound must be introduced; cf. Schröger and Wolff, 1996). MMN can also be recorded in non-human species (e.g., Javitt et al., 1994; Tikhonravov et al., 2008) including rodents (Astikainen et al., 2006; Nakamura et al., 2011; Budd et al., 2012; Shiramatsu et al., 2013).

Because the detection of “novelty” (i.e., simple acoustic deviance or complex regularity violation) requires information storage and comparison over time, it must involve more or less complex memory operations (Näätänen et al., 2001, 2010; Winkler, 2007). For this reason it is commonly believed that novelty detection must be accomplished at the level of the cortex. However, the fact that the MMN persists during sleep or anesthesia suggests that it is “preattentive” in origin (Tiitinen et al., 1994) and therefore could originate subcortically. Although this idea received support from early seminal studies suggesting subcortical generators to the MMN (Kraus et al., 1994; Csépe, 1995), it has been largely unexplored until recently (Escera and Malmierca, 2014). Yet, recent human studies have suggested that subcortical auditory stations may undergoo substantial experience-dependent plasticity (Chandrasekaran and Kraus, 2010; Kraus and Skoe, 2010; Chandrasekaran et al., 2013; Skoe et al., 2013, 2014). For example, Kraus et al. have found that the brainstem activity is enhanced when stimulus sequences contain predictable sounds compared to more random (i.e., unpredictable) sequences that is infrequent and unpredictable. In the present perspective, we will present data to suggest the active role of the inferior colliculus (IC), and in general of the subcortical auditory pathway in regularity encoding and deviance detection”. It is even suggested that the IC is analogous to V1 in processing complexity (King and Nelken, 2009). Yet the assumption that novelty detection is a cortical function has persisted, not only for theoretical reasons (complexity of the involved regularities as explained above), but also for technical reasons, because it is difficult to pinpoint the site at which scalp EEG waveforms are generated, especially in the case of subcortical structures.

Importantly, over the past 10 years, a similar phenomenon to that described for MMN has been demonstrated to occur at the cellular level using neurophysiological tools. Single neuron recordings using an oddball paradigm similar to that used for MMN studies have shown a decreased response to a repeated (standard) sound and an increased response to a less repeated (deviant) sound within a sound sequence in the cat auditory cortex (AC; Ulanovsky et al., 2003). This phenomenon has been termed “stimulus-specific adaptation” (SSA) and was originally proposed as the neuronal correlate of MMN. Moreover, it was assumed that SSA was a unique and emerging property of the AC neurons (Ulanovsky et al., 2003; Nelken and Ulanovsky, 2007).

**Figure 1:**
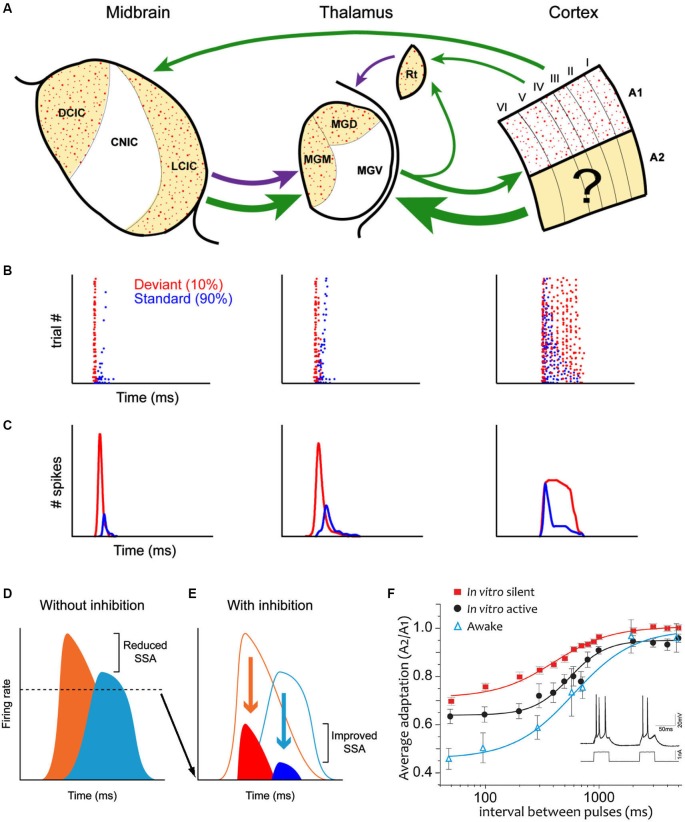
**(A)** *Schematic* diagram (after Escera and Malmierca, 2014) illustrating the main anatomical subdivisions as well as the similarities and differences of SSA at the IC (left column), MGB (middle column) and auditory cortex (AC) (right column). Arrows indicate the major connections between these regions. Green arrows are excitatory projections, purple arrows inhibitory connections. Non-lemniscal divisions are highlighted as yellow-shade areas and stipple areas show regions where SSA is strong. Note that SSA is linked to non-lemniscal regions in IC and MGB but to the lemniscal primary AC. Dot raster plots **(B)** and peri-stimulus histogram (PSTHs); **(C)** that show the adaptation of the response to the standard stimulus (blue dots) while the response to the deviant stimulus (red dots) does not adapt. **(D)** Neurons respond to deviants (orange) and standards (light blue) with high firing rates, in the absence of inhibition and thus the deviant to standard ratio is small. By contrast, GABA_A_- mediated inhibition **(E)** reduces the responses to both deviants (red) and standards (dark blue) acting as in the “iceberg effect” increasing the deviant to standard ratio and thus enhancing SSA. For more details see Pérez-González et al. (2012). **(F)** Average adaptation time course in single neurons in the AC in the awake animal, in silent cortical slices and “active” cortical slices (where the intracellularly recorded neuron was induced to fire following a prerecorded neuron in the awake animal). Note that all adaptation *in vitro* is due strictly to cellular (and not synaptic) mechanisms. To estimate the time course, two identical 50 ms pulses of current injection were delivered with intervals spanning from 50 ms to 5 s (see inset). The relative frequency rate of the second response with respect to the first one are represented. Note that while the time course is similar, the larger adaptation is that in the awake animal and the least the one in the silent slice. Part of the difference between those two is due to the ongoing activity in the awake, as the *in vitro* “active” preparation indicates. A logistic function was fitted. Error bars are SEM. For more details see Abolafia et al. (2011). Abbreviations: A1, primary auditory cortex; A2 non-primary auditory cortex; CNIC, central nucleus of the inferior colliculus; DCIC, dorsal cortex of the inferior colliculus; LCIC, lateral cortex of the inferior colliculus; MGD, dorsal division of the medial geniculate body; MGM, medial division of the medial geniculate body; MGV, ventral division of the medial geniculate body; Rt, reticular thalamic nucleus.

However, there is now a substantial body of evidence challenging this idea (Pérez-González et al., 2005, 2012; Anderson et al., 2009; Malmierca et al., 2009; Yu et al., 2009; Antunes et al., 2010; Antunes and Malmierca, 2011, 2013; Bäuerle et al., 2011; Zhao et al., 2011; Duque et al., 2012, 2014; Patel et al., 2012; Ayala et al., 2013; Ayala and Malmierca, 2013; Pérez-González and Malmierca, 2014). These studies have demonstrated that SSA also occurs subcortically (Figure 1A), i.e., in the IC and medial geniculate body (MGB). Moreover, many aspects of SSA seen in the IC (Ayala and Malmierca, 2013; Pérez-González and Malmierca, 2014) and in the MGB (Antunes et al., 2010; Antunes and Malmierca, 2013) are very similar (Figures 1B,C) to that described in the AC (Ulanovsky et al., 2003, 2004; von der Behrens et al., 2009; Taaseh et al., 2011). However, a major difference between the cortical and subcortical SSA is that SSA in the IC and MGB is stronger in the non-lemniscal divisions, while the first lemniscal nucleus where SSA is strong and widespread is the primary AC (Ulanovsky et al., 2003; Nelken and Ulanovsky, 2007). A simple interpretation would be that SSA is merely imposed upon IC and MGB neurons (Nelken and Ulanovsky, 2007) through the massive descending corticofugal pathways in a top-down fashion (Malmierca and Ryugo, 2011). However, recent work has demonstrated that SSA in the MGB and IC is not merely inherited from the AC (Antunes and Malmierca, 2011; Anderson and Malmierca, 2013) since cortical deactivation does not change SSA sensitivity. Hence SSA may be created independently at each level of the auditory hierarchy (Escera and Malmierca, 2014). For example, the pharmacological manipulation of GABA_A_ receptors in the IC (Pérez-González et al., 2012; Pérez-González and Malmierca, 2012) and MGB (Duque et al., 2014) has shown that, while not involved in the generation of SSA, inhibitory inputs could modulate the level of adaptation by reducing the relative strength of the response to the standard and deviant stimuli increasing the deviant to standard ratio, acting as a gain control mechanism, similar to the iceberg effect (Figures 1D,F; Pérez-González et al., 2012). Note that this does not exclude the possibility that the consequences of SSA are also transmitted to the next relay station as in a cascade. In fact, adaptation effects cascade through the visual system (Dhruv and Carandini, 2014).

## Cellular mechanisms of auditory cortex adaptation

The previous section outlined findings showing that auditory adaptation (often referred to as forward masking/suppression; e.g., Wehr and Zador, 2005; Scholes et al., 2011) can be attributed not only to AC but also to subcortical structures (cf. Pérez-González and Malmierca, 2014). Besides this anatomical diversity, adaptation shows diversity as well in the temporal dimension: it occurs at different time scales spanning many orders of magnitude, from several milliseconds to tens of seconds (Ulanovsky et al., 2004; Costa-Faidella et al., 2011). This broad time range makes it compatible with the participation of different potential underlying mechanisms. Some of these are synaptic mechanisms such as: synaptic depression (Wehr and Zador, 2005), decreased excitation or lateral inhibition (Qin and Sato, 2004), increased inhibition (Zhang et al., 2003), or excitatory/inhibitory imbalance (De Ribaupierre et al., 1972; Volkov and Galazjuk, 1991; Ojima and Murakami, 2002; Oswald et al., 2006).

Paradoxically, the role of intrinsic membrane mechanisms such as potassium currents in AC adaptation had been ruled out or neglected until recently, even though they participate in sensory adaptation in other sensory cortices: sensorimotor (Schwindt et al., 1988), barrel (Diaz-Quesada and Maravall, 2008), or visual cortex (Sanchez-Vives et al., 2000a,b; Wang et al., 2003). Potassium currents act as an activity-dependent adaptation mechanism, such that depolarization and high frequency firing during sensory responses induce not only the activation of voltage-dependent potassium currents but also an intracellular increase of ions like Ca^2+^ and Na^+^ that activate ion-dependent K^+^ channels (for reviews see Sah and Faber, 2002; Bhattacharjee and Kaczmarek, 2005). Even if a synaptic depolarization is subthreshold, sodium entering through glutamate receptors can activate sodium-dependent K^+^ channels (Nanou et al., 2008). The activation of potassium currents hyperpolarizes the membrane potential, decreasing the neuronal responsiveness to subsequent inputs with time courses that range from tens of milliseconds to tens of seconds. Such slow time courses are supported by the dynamics of the relevant intracellular ions –in particular Na^+^– in the vicinity of K^+^ channels and their binding/unbiding to them. Sodium and calcium-dependent potassium currents also exist in neurons of the rat AC (Abolafia et al., 2011).

In order to explore whether potassium channels do actually play a role in auditory adaptation in the awake animal, it was first necessary to determine the time course of adaptation of AC neurons in chronically implanted awake rats. In a recent study, Abolafia et al. (2011) delivered two auditory stimuli separated by intervals ranging between milliseconds and several seconds. The attenuation of the response to the second stimulus with respect to the first along time provided the time course of adaptation, showing that a 50 ms sound in the awake animal influences responses occurring up to 2–5 s later. The same auditory protocol was then mimicked in the cortical neurons *in vitro* (Figure 1F) through intracellular current injections that had the same duration and would evoke the same number of spikes as the auditory stimuli did in the awake animal. It should be noted that those were silent slices and therefore all the observed phenomena only involve the intracellularly recorded neuron and not the network in which it is embedded. This approach demonstrated that the time course of adaptation observed in the awake rat was highly similar to the one detected *in vitro* (Abolafia et al., 2011). However, the response attenuation was larger in the awake animal. A critical factor contributing to this difference is the spontaneous ongoing activity in the awake animal, that builds up adaptation currents contributing to a steady state of adaptation in an active (awake) cortical network with respect to a silent one (slice). This steady state of adaptation has been demonstrated by replicating spontaneous firing recorded from neurons in the awake animal in *in vitro* neurons by means of intracellular current injection (Figure 7; Abolafia et al., 2011).

Therefore, a significant fraction of cortical auditory adaptation can be explained just by intrinsic cellular mechanisms (K^+^ channels), although this does not exclude the additional participation of synaptic mechanisms. Hence ionic mechanisms should be incorporated into mechanistic explanations of adaptation and novelty detection. Further, the interaction between potassium channels-mediated adaptation and synaptic depression provides computational capabilities to the network to detect rate of change, anticipation and detection of novelty (Puccini et al., 2006, 2007).

An argument that is often used against ionic channels as a mechanism for auditory adaptation is that they lack input specificity and thus they would attenuate any incoming input, not supporting the delicate SSA (Ulanovsky et al., 2003, 2004; Wehr and Zador, 2005). However, based on cortical circuitry, a speculation would be that cellular hyperpolarization and decreased excitability are cellular properties that become network properties by reverberating in the local recurrent circuitry of the cortical column, contributing to adaptation in neurons with the same frequency-specificity.

## Novelty detection in a cortico-subcortical distributed cerebral network

**Figure 2:**
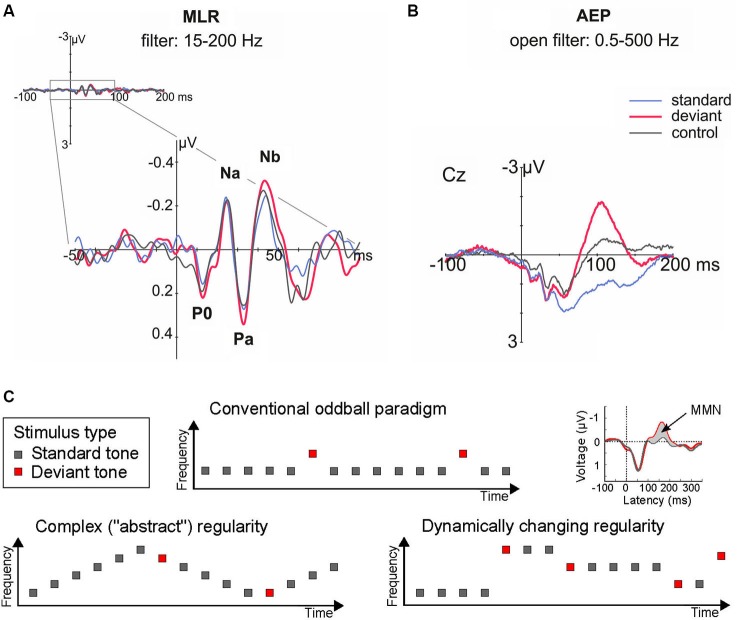
**(A–B)** Human auditory evoked potentials in the latency range of the middle latency response (MLR, at circa 20–50 ms; **A**, left), and later on, by the long latency response (circa 100–200 ms; **B**, right), reveal that deviance detection based on regularity encoding takes place in human AC at recurrent neural networks; red, deviant response; blue, standard response; black, control response. Note that physically identical sounds elicited larger responses when they were presented as deviant than as standard or control stimuli in the MLR (by the Nb component, at 40 ms from change onset) and long-latency (i.e., MMN, by 100 ms) ranges. Adapted from Grimm et al. (2011). **(C)** Schematic illustration of stimulus paradigms departing from the standard oddball paradigm to achieve an increase in complexity and ecological validity. These and similar paradigm variations have shown that complex regularities in the acoustic environment can be extracted from just a few exemplars, as demonstrated by long-latency auditory evoked potentials (the MMN).

The idea that novelty detection and the underlying regularity encoding can take place at levels hierarchically lower/earlier than those generating the MMN, is also supported by a series of human studies that recorded middle latency responses (MLRs) in the oddball paradigm. The MLR is a series of characteristic waveforms elicited to discrete auditory stimuli in the range 12–50 ms post-onset (Figure 2A). They are labeled as N0, P0, Na, Pa, and Nb (sometimes Pb, equivalent to P50, is included), and represent the earliest cortical responses to a sound (Winkler et al., 2013). For example, it has been shown that the P0 waveform peaking at 16–19 ms is generated in primary AC, whereas subsequent components are generated in surrounding areas of the supratemporal plane and gyrus (Yvert et al., 2001, 2005).

One seminal study investigating novelty detection in the MLR time range (Grimm et al., 2011) used a frequency “deviant” tone of 1200 Hz presented amongst 800 Hz standard tones, and implemented a “reversed” condition (where deviant and standard switched their roles, controlling for stimulus-feature effects), and a “controlled” condition (where the deviant was embedded amongst a series of other equiprobable tones, controlling for refractoriness effects; cf. Schröger and Wolff, 1996). The results (Figure 2A) showed an enhanced Nb MLR component elicited by the deviant tones when compared both to the reversed standard and to the control stimulus. This reveals that “true” deviance detection (i.e., based on regularity encoding rather than simple adaptation) occurs at latencies as short as 40 ms from sound onset (Grimm et al., 2011). This demonstration of regularity-based deviance detection is important to develop a general framework encompassing not only simple adaptation phenomena (i.e., based on stimulus repetition; cf. Grill-Spector et al., 2006), but also more complex forms of auditory regularity encoding.

Subsequent studies confirmed the involvement of the Nb in frequency deviant detection (Alho et al., 2012; Leung et al., 2012; Althen et al., 2013), and revealed that other MLR waveforms are related to deviant detection in other auditory stimulus features, such as the Na for location changes (Sonnadara et al., 2006; Cornella et al., 2012; Grimm et al., 2012), and the transition between the Na-Pa waveforms for intensity changes (Althen et al., 2011) or the Pa-Nb for temporal deviations (Leung et al., 2013).

A further magnetoencephalographic (MEG) study revealed that deviant-detection is in fact a distributed property of the AC, with neural populations generating its Nb/MLR correlates located in more medial and anterior regions than those giving rise to the MMN (Recasens et al., 2014a). Hence, these early deviance-related responses by the MLR (Figure 2A) may represent, by their earlier latency and lower hierarchical distribution, a better human psychophysiological correlate of the single-unit novelty responses than the MMN (Figure 2B; Escera et al., 2013).

At lower auditory stations, while no correlates of deviance detection were found in the auditory brainstem response (ABR; Slabu et al., 2010; Althen et al., 2011), a recent study that measured the frequency following response (FFR; Skoe and Kraus, 2010) revealed the involvement of the human IC in deviance detection based on regularity encoding (Slabu et al., 2012) as in animal studies (Gao et al., 2014).

Interestingly, studies addressing whether more complex types of auditory regularities could be encoded at the level of the MLR-generating system, such as tone alternation (Cornella et al., 2012), feature conjunction (Althen et al., 2013) or pattern regularities (Recasens et al., unpublished observations), yielded negative results. These negative findings, together with the early effects described above for simple feature violations, suggest that the auditory system is organized in a hierarchical fashion, so that complex regularities require higher levels of the auditory hierarchy to be picked up (Grimm and Escera, 2012; Escera and Malmierca, 2014). However, it should be noted that preliminary studies using single unit recordings in animals suggest that IC neurons show sensitivity to complex regularities (Aguillon et al., 2013).

## Regularity encoding and novelty detection in complex stimulus environments

Now that the existence of “novelty responses” along the auditory pathway is well established both in animal and human studies, an important next step will be to characterize the properties of this mechanism in more detail—particularly with more ecologically valid stimulus configurations (Figure 2C). Initial demonstrations of these effects have often used repetitive sound sequences over long recording periods, making it difficult to distinguish between simple (repetition-based) adaptation and a “true” regularity encoding account. But realistic sound sources exhibit more complex forms of organization; for instance, they do not repeat their sound emissions but change them in a regular manner (e.g., when gradually moving to a different location). There is now abundant evidence to show that the MMN responds to violations of such complex forms of regularities (for reviews, see Näätänen et al., 2001, 2010; Winkler, 2007). Whether the same is true at earlier levels of the auditory hierarchy is still a matter of investigation (see above; cf. Cornella et al., 2012; Aguillon et al., 2013; Althen et al., 2013). It is important to resolve this issue to develop better links between the neuronal models reviewed above and theoretical accounts of regularity encoding coming from MMN-based investigations (Winkler, 2007) or from closely related research fields such as predictive coding (Friston, 2005).

Another form of real-life complexity has received much less attention: a sound source usually does not exhibit the same type of regular behavior over hours, but behaves according to one regularity for a while and then according to another regularity (e.g., it stands still, gradually changes location, then stands still again). Hence the brain should be able to extract regularities considerably faster than shown in a typical protocol where the same regularity is valid for an hour or longer. Some MMN studies have demonstrated that regularity extraction is possible from just a few exemplars (Cowan et al., 1993; Winkler et al., 1996; Huotilainen et al., 2001; Haenschel et al., 2005; Bendixen et al., 2007, 2008). Both human and animal data suggest that the brain picks up regularities at multiple timescales (Ulanovsky et al., 2004; Costa-Faidella et al., 2011). This is important because it demonstrates quick adaptation to newly emerging regularities as well as longer-lasting impact of previously valid regularities, both of which appear to be ecologically adaptive. Systematic investigations along these lines across different levels of the auditory hierarchy should prove informative for a comprehensive model of regularity encoding and novelty detection.

Characterizing the mechanisms and temporal dynamics of regularity encoding is important also in the face of its implications for other cognitive processes. Sensory regularities render the environment predictable and hence serve preparation of appropriate motor responses. Another benefit of regularity encoding may lie within perception itself: Regularities have been shown to support auditory scene analysis, i.e., disentangling a mixture of overlapping signals emitted by several concurrently active sound sources (see Bendixen, 2014, for review). Furthermore, the ability to use certain regularities for sound source segregation appears to decline with age (Rimmele et al., 2012), possibly linked with an age-related decline in regularity encoding capacities (Pekkonen, 2000; Näätänen et al., 2012). Hence a comprehensive understanding of regularity encoding and novelty detection, as well as of impairments in these processes, may shed light on crucial aspects of everyday listening experience.

## Concluding remarks

In the auditory system, the sequence of previous sensory stimulation and its resulting neuronal activation deeply influence subsequent responses—providing the ground for auditory adaptation, regularity encoding, and novelty detection. The anatomical stages and mechanisms contributing to these phenomena can now be partly delineated based on recent advances outlined above. Animal studies help to shed light on the neurophysiological mechanisms subserving adaptation. We have argued that not only synaptic but also cellular mechanisms should be taken into account as viable contributors. Furthermore, we suggested that it may be more appropriate to link SSA with MLR deviance-related effects than with the MMN component.

Human studies are contributing to a more comprehensive picture of the mechanisms involved in auditory perception where regularity encoding seems to be paramount. Moreover, human ERP studies provide a link to clinical neuroscience so that pathophysiological mechanisms of auditory information processing in neurological, psychiatric and developmental disorders can be revealed (Näätänen et al., 2011, 2012). In this regard, the recently discovered correlates of auditory deviance detection by the MLR latency range, and even the psychophysiological subcortical correlates revealed by the FFR may help to go a step beyond the MMN in understanding these disorders. Animal correlates again provide a valuable complementary approach to elucidate the role of novelty and adaptation in auditory perception under normal and pathological conditions. Additional insight may be gained by studying animal models in the same way as done in human studies (e.g., MLR, MMN). With this perspective article, we hope to contribute to further promising research at this fascinating intersection.

## Conflict of interest statement

The authors declare that the research was conducted in the absence of any commercial or financial relationships that could be construed as a potential conflict of interest.
